# 급성약물중독으로 응급의료센터를 내원한 자살시도자의 자살 재시도 영향요인: 후향적 연구

**DOI:** 10.7586/jkbns.24.014

**Published:** 2024-08-20

**Authors:** In Ah Yun, Mi Yang Jeon

**Affiliations:** 1 Gyeongsang National University Changwon Hospital, Chanwon, Korea; 1 창원경상국립대학교병원; 2 College of Nursing, Sustainable Health Research Institute, Gyeongsang National University, Jinju, Korea; 2 경상국립대학교 간호대학 간호학과·지속가능건강연구소

**Keywords:** Drug-related side effects and adverse reactions, Retrospective studies, Suicide, attempted, 약물의 부작용 및 이상반응, 후향적연구, 자살시도

## Abstract

**Purpose:**

This retrospective study was conducted to identify factors associated with repeated suicide attempts by analyzing the electronic medical records of suicide attempters who visited emergency medical centers due to drug intoxication.

**Methods:**

Data from January 1, 2020 to December 31, 2021 were collected for this study and analyzed using SPSS/WIN 25.0.

**Results:**

In this study, the factors affecting repeated suicide attempts among individuals who visited the emergency medical center due to acute drug intoxication were the presence or absence of psychiatric diseases, the season of drug intoxication, and age. In order to identify the factors affecting repeated suicide attempts in those who presented with acute drug intoxication, logistic regression analysis was performed by inputting psychiatric diseases, season of drug intoxication, and age, all of which exhibited significant differences between those who did and did not attempt suicide again. The presence of psychiatric diseases (odds ratio [OR] = 1.99, 95% confidence interval [CI] = 1.04~3.80), intoxication occurring in the fall (vs. spring; OR = 3.35, 95% CI = 1.28~8.75), and age ≥ 65 (vs. those under the age of 18; OR = 12.96, 95% CI = 2.02~83.17). The regression model was found to be statistically significant (F = 30.01, *p* < .001), and the coefficient of determination of Cox and Snell, which represents the explanatory power of the model, was 12.0%.

**Conclusion:**

This study suggests that to prevent repeated suicide attempts, it is necessary to develop a tool to evaluate the risk of suicide, use it to evaluate the risk of suicide, and then manage individuals accordingly.

## 서론

### 1. 연구의 필요성

우리나라 자살률은 2018년부터 2022년 현재까지 경제협력개발기구(Organization for Economic Co-operation and Development) 국가 중 1위를 차지하고 있으며 자살은 암, 심장질환, 폐렴, 뇌혈관질환에 이어 사망원인 5위이다[1]. 자살시도자 중 약 37%가 적어도 한 번 자살을 재시도하며, 자살시도의 10%가 10년 이내 자살로 인한 사망으로 이어진다[2]. 또한 자살 1회 시도자보다 자살 재시도자가 치명적인 자살을 시도하는 경우가 많으므로[3] 자살시도자의 자살 시도 원인과 자살 재시도의 원인을 파악할 수 있다면 자살을 예방하는데 중요한 자료가 될 수 있을 것이다.

2020년 자살시도자의 자살 방법을 살펴보면 목맴이 가장 많으며, 그 다음은 추락, 가스 중독, 농약 중독이며, 전년 대비 증가율이 가장 높은 것은 급성약물중독이었다[4]. 급성약물중독은 고의나 실수로 치료약물 또는 물질을 과량 복용하여 약물 본래의 효과 이외의 독성작용이 발생하는 상태이다. 자살을 목적으로 의도적으로 약물을 복용하는 급성약물중독에는 치료 약물과 농약이 가장 높은 비율을 차지하였다[5]. 급성약물중독은 중독 물질의 종류와 양, 중독 후 응급의료센터까지 소요 시간에 따라 치료가 달라지며, 치료 후에도 심각한 후유증과 합병증을 초래할 수 있어 보호자 및 환자에게 경제적 및 정신적 문제를 유발할 수 있다[6]. 급성약물중독으로 응급실을 내원한 환자의 비율은 3.0%이다[7]. 급성약물중독으로 응급실을 내원한 환자는 남자가 36.9%, 여자는 63.1%이며, 연령은 20대가 23.3%로 가장 많으며 다음은 60세 이상이 21.7%, 10대 13.7%이었고, 중독 약물로는 치료약물 77.9%, 농약 9.3%, 가스 7.0%, 인공독성물질 5.4%이었다[8]. 급성약물중독으로 자살을 시도하는 대상자의 30% 이상이 재시도하고[9,10], 급성약물중독으로 인한 자살시도자의 자살 진정성을 평가한 연구[11]에서 대상자들의 70.7%는 도움을 얻으려고 했던 것이지, 정말 죽으려고 했던 것은 아니라고 응답하였으며, 정말 죽으려고 그럴만한 방법을 선택했다고 응답한 비율은 12.1%로 대다수의 자살시도자가 도움을 요청하기 위해 시위 수준의 진정성으로 자살을 시도하는 것으로 보고하였다. 급성약물중독으로 자살을 시도하는 환자들이 복용하는 약물은 주로 수면제, 항우울제 등 질환을 치료하기 위해 처방받은 약물[12]이기 때문에 자살 시도 이후에도 약물을 지속적으로 복용할 가능성이 높으므로 급성약물중독으로 자살을 시도한 환자의 자살 재시도를 예방하는 것이 필요하다.

자살시도자의 대부분은 응급실에 내원하여 응급처치를 받은 후 자의 퇴원하거나 중환자실이나 병실로 입원하기 때문에[6] 입원 초기단계에서부터 자살시도자를 관리하기 위해서는 응급실 간호사의 역할이 중요하다[13]. 그러나 자살시도자를 간호하는 응급실 간호사들은 자살시도자 간호에 대해 혼란을 경험하고 있으며 전문적인 준비가 부족하여 불충분한 간호를 제공하는 것에 대한 죄책감을 가지고, 전문가적인 접근이 필요하다고 인식하고 있었다[12]. 최근 응급의료센터에 내원한 자살시도자와 관련된 연구[10]에서는 자살시도자의 특성을 분석한 연구와 응급실 기반 자살시도자 사후관리 사업 참여에 영향을 미치는 요인[14,15] 또는 사후관리사업을 완수한 자와 완수하지 못한 자의 차이를 비교한 연구[16]가 있었으며, 일부 선행연구[17,18]에서 응급실에 내원한 자살시도자의 자살 재시도에 영향을 미치는 요인은 결혼, 정신질환, 생활 사건에서 대인관계 갈등, 정신건강의학과적 문제, 자살 시도 전후 도움 요청, 항우울제 복용 등으로 보고하였다. 그러나 자살시도자의 자살 재시도를 예방하기 위해서는 자살시도자들이 자살을 재시도하는데 영향을 미치는 요인을 규명하는 것이 필요하다.

이에 본 연구에서는 응급의료센터에 급성약물중독으로 내원한 자살 1회 시도자와 자살 재시도자 간의 특성을 비교 분석하고, 자살 재시도에 영향을 미치는 요인을 규명하여 급성약물중독으로 자살을 시도하고 응급의료센터를 내원한 자살시도자의 자살 재시도를 방지할 수 있는 관리 프로토콜을 개발하는데 기초 자료로 제공하고자 한다.

### 2. 연구 목적

본 연구의 목적은 급성약물중독으로 응급의료센터를 내원한 자살시도자의 자살 재시도에 영향을 미치는 요인을 규명하는 것이며, 구체적인 목적은 다음과 같다.

1) 급성약물중독으로 응급의료센터를 내원한 자살시도자 중 자살 1회 시도자와 자살 재시도자 간의 일반적 특성, 급성약물중독, 중증도 관련 특성, 치료 관련 특성 관련 특성을 비교한다.

2) 급성약물중독으로 응급의료센터를 내원한 자살 재시도자의 자살 재시도에 영향을 미치는 요인을 규명한다.

## 연구 방법

### 1. 연구 설계

본 연구는 급성약물중독으로 응급의료센터를 내원한 자살 1회 시도자와 자살 재시도자의 일반적 특성, 급성약물중독 관련 특성, 중증도 관련 특성, 치료 관련 특성을 비교하고, 자살 재시도에 영향을 미치는 요인을 규명하기 위한 후향적 조사연구이다.

### 2. 연구 대상

본 연구의 대상자는 2020년 1월 1일∼ 2021년 12월 31일까지 창원경상국립대학교병원 응급의료센터에 급성약물중독으로 내원한 자살시도자를 대상으로 하였으며, 대상자의 선정 기준 중 포함 기준은 과거 자살 시도력을 확인할 수 있는 자이고, 제외 기준은 비의도적으로 약물을 복용한 자이다. 본 연구의 대상자는 연구기간 동안 급성약물중독으로 응급의료센터를 내원한 환자 총 416명이었으며 이중 자살을 목적으로 의도적으로 약물을 복용한 자는 290명이었다. 이 중 과거 자살시도에 대한 정보를 확인할 수 있는 195명을 대상으로 하였으며, 자살 1회 시도자는 125명, 자살 재시도자는 70명이었다.

### 3. 연구 도구

#### 1) 급성약물중독 조사지

본 연구에서 사용한 급성약물중독 조사지는 미국 중독관리센터협회(the American Association of Poison Control Centers)의 독성노출조사체계(Toxic Exposure Surveillance System) [19]를 대한임상독성학회[20]에서 한국 실정에 맞게 수정하고, 국문화한 조사지와 급성약물중독 관련 문헌고찰을 근거로 본 연구자가 개발하였다. 본 연구에서 개발한 급성약물중독 조사지의 전문가 내용타당도는 간호학 교수 2인, 응급의료센터 간호사 2인, 응급의학과 전문의 1인에게 문항별 내용타당도를 검증받으며, 문항별 전문가 내용타당도는 .80이상이었다. 본 연구에서 개발한 급성약물중독 조사지는 일반적 특성 5개 문항, 약물 중독 관련 특성 9개 문항, 중증도 관련 특성 3개 문항, 치료 관련 특성 6개 문항, 자살 재시도 관련 특성 2개 문항을 포함하여 총 25개 문항으로 구성되었다. 급성약물중독 조사지의 구체적인 내용은 다음과 같다.

##### (1) 일반적 특성

본 연구에서 일반적인 특성은 성별, 연령, 동거가족 유무, 직업 유무, 기저질환을 포함한 5개 문항으로 구성하였다.

##### (2) 약물중독 관련 특성

본 연구에서 급성약물중독 관련 특성은 의도성 여부, 의도적 급성약물중독 원인, 중독 물질, 중독 장소, 중독 계절, 중독 일시, 응급실 내원 일시, 내원수단, 내원 경로, 과거 자살시도 유무, 과거 자살시도 횟수를 포함한 11개 문항으로 구성하였다.

##### (3) 중증도 관련 특성

본 연구에서 중증도 관련 특성은 내원 시 의식상태, 활력징후, 중증도 등급(Korean Triage and Acuity Scale, KTAS) [21]을 포함하여 3개 문항으로 구성하였다.

##### (4) 치료 관련 특성

본 연구에서는 치료 관련 특성은 중독 치료, 치료 결과, 혈액검사, 방사선검사, 심전도 검사, 독성물질 검사를 포함한 6개 문항으로 구성하였다.

혈액검사 13개 항목은 백혈구(white blood count or cell, WBC), 적혈구(red blood count or cell, RBC), 호중구 수치(absolute neutrophil count, ANC), C-반응성 단백질(C-reactive protein, CRP), 알칼리인산분해요소(alkaline phosphatase, ALP), 아스파테이트 아미노전이효소(aspartate aminotransferase, AST), 간-효소수치(alanine aminotransferase, ALT), 감마-글루타밀전이효소(gamma-glutamyl transferase, GGT), 크레아틴키나제(creatine kinase, CK), 크레아틴키나아제(creatine kinase–muscle brain, CK-MB), 뇌나트륨이뇨펩티드(N-terminal pro b-type natriuretic peptide, NT-proBNP), 혈액요소질소(blood urea nitrogen, BUN), 크레아티닌(creatinine)으로 구성하였다. 방사선 검사는 Chest X-ray로 확인하였고, 결과는 ‘정상’, ‘비정상’과 ‘시행하지 않음’ 으로 분류하였다. 심전도 검사는 표준 12유도 심전도로 검사하였으며, 결과는 ‘정상’, ‘비정상’과 ‘시행하지 않음’ 으로 분류하였다. 독성물질 검사는 소변을 이용하여 11종 마약 및 독성물질을 분석하였다. 독성물질 검사를 위해 소변을 채취할 때는 멸균적으로 채취하기 위해 nelaton 또는 foley catheter를 삽입하여 채취하였다.

### 4. 자료 수집

의무기록실에 2020년 1월 1일~2021년 12월 31일까지 경상대학병원 응급의료센터에 급성약물중독으로 내원한 자살시도자의 일반적 특성, 급성약물중독 관련 특성, 중증도 관련 특성, 치료 관련 특성에 대한 자료를 요청하여 수집하였다.

### 5. 자료 분석

본 연구에서 수집한 자료는 SPSS statistics software(version 25.0; IBM., Armonk, NY, USA)을 이용하여 분석하였으며, 구체적인 분석방법은 다음과 같다.

1) 자살 1회 시도자와 재시도자 간의 일반적 특성, 급성약물중독 관련 특성, 중증도 관련 특성, 치료 관련 특성의 차이는 평균, 표준편차, Chi-square test, Fisher’s exact test, independent t-test를 이용하여 분석하였다.

2) 자살 재시도에 영향을 미치는 요인은 로지스틱 회귀분석(Logistic Regression)으로 분석하였다.

### 6. 윤리적 고려

본 연구는 경상대학교 병원 임상시험심사위원회(IRB File No. 2022-02-018)의 승인을 받은 후 의무기록실에 응급의료센터에 내원한 급성약물중독환자의 정보를 요청하였다. 의무기록실에서 제공받은 대상자의 개인 정보는 1차적으로 암호화하여 자료를 취합하였고, 연구를 위해 수집된 자료와 개인 정보는 개별화된 ID를 부여하고 코드화하여 비밀을 보호하였다. 수집한 자료는 생명윤리법 시행규칙 제15조에 따라 연구가 종료된 시점인 2022년 1월 1일부터 환자정보 조사지와 자료를 저장한 USB는 3년간 잠금장치가 있는 서랍에 보관한다. 환자정보 조사자와 USB는 보관기간이 지나면 개인정보보호법 시행령 16조에 따라 폐기할 예정이다.

## 연구 결과

### 1. 자살 1회 시도자와 자살 재시도자의 일반적인 특성의 차이

본 연구에서 응급의료센터에 급성약물중독으로 내원한 자살시도자는 195명이며, 자살 1회 시도자는 125명(64.1%), 자살 재시도자는 70명(35.9%)이었다. 자살 1회 시도자와 자살 재시도자의 일반적 특성의 차이 중 성별(χ^2^ = 2.28, *p* = .131), 연령(t = −2.83, *p* = .625), 동거가족 유무(χ^2^ = 0.99, *p* = .319), 직업 유무(χ^2^ = 0.26, *p* = .609)는 두 군간에 통계적으로 유의한 차이가 없었다.

본 연구 대상자의 기저질환을 살펴보면, 정신건강의학과 질환은 자살 1회 시도자는 53명(42.4%), 자살 재시도자는 43명(61.4%)으로 두 군간 통계적으로 유의한 차이가 있었으나(χ^2^ = 6.50, *p* = .011), 기저질환 중 고혈압(χ^2^ = 0.02, *p* = .884), 당뇨병(χ^2^ = 1.15, *p* = .284), 결핵(*p* = .641), 간질환(*p* = .359)과 심장질환(χ^2^ = 2.28, *p* = .130)은 군간에 유의한 차이가 없었다(Table 1).

### 2. 자살 1회 시도자와 자살 재시도자의 급성약물중독 관련 특성의 차이

자살 1회 시도자와 자살 재시도자의 급성약물중독 관련 특성의 차이는 Table 2와 같다. 본 연구에서 자살시도 이유를 살펴보면, 자살 1회 시도자는 심리적 문제(우울, 불안)가 48명(38.4%)로 가장 많았으며, 다음은 가족 문제 40명(32.0%), 경제적 문제 23명(18.4%), 질병 비관 9명(7.2%), 지인과 갈등 4명(3.2%), 기타 1명(0.8%) 순이었다. 자살 재시도자는 심리적 문제가 34명(48.6%)으로 가장 많았으며, 다음은 가족 문제 16명(22.9%), 경제적 문제 10명(14.3%), 질병 비관 6명(8.6%), 기타 3명(4.3%), 지인과 갈등 1명(1.4%)으로 나타나 두 군간 통계적으로 유의한 차이가 없었다(χ^2^ = 6.17, *p* = .290).

본 연구에서 자살시도 약물을 살펴보면, 자살 1회 시도자는 항우울제가 45명(36.0%)으로 가장 많았으며, 다음은 수면제 35명(28.0%), 제초제 25명(20.0%), 진통제 8명(6.4%), 살충제 6명(4.8%), 기타 5명(4.0%), 미상 1명(0.8%) 순이었다. 자살 재시도자는 항우울제가 30명(42.9%)으로 가장 많았으며, 수면제 27명(38.6%), 제초제 7명(10.0%), 진통제 4명(5.7%), 살충제와 미상이 각 1명(1.4%)으로 나타나 두 군간 통계적으로 유의한 차이가 없었다(χ^2^ = 9.29, *p* = .158).

본 연구에서 급성약물중독 계절을 살펴보면, 자살 1회 시도자는 가을(9∼11월)이 42명(33.6%)으로 가장 많았으며, 다음은 여름(6∼8월)이 31명(24.8%)이었고, 봄(3∼5월)이 27명(21.6%), 겨울(12∼2월)이 25명(20.0%)이었다. 자살 재시도자는 겨울(12∼2월)이 22명(31.4%)으로 가장 많았으며, 다음은 여름(6∼8월)이 20명(28.6%), 봄(3∼5월)이 18명(25.7%)이었고, 가을(9∼11월) 10명(14.3%)으로 나타나 두 군간 유의한 차이가 있었다(χ^2^ = 9.28, *p* = .026).

본 연구에서 급성약물중독으로 자살을 시도한 시간(χ^2^ = 1.98, *p* = .576)간, 자살을 시도하고 응급의료센터를 내원한 시간(χ^2^ = 1.60, *p* = .659), 응급의료센터 내원수단(χ^2^ = 1.31, *p* = .519)과 응급의료센터를 내원한 경로(χ^2^ = 0.82, *p* = .663)는 두 군간 통계적으로 유의한 차이가 없었다(Table 2).

### 3. 자살 1회 시도자와 자살 재시도자의 중증도 관련 특성의 차이

자살 1회 시도자와 자살 재시도자의 중증도 관련 특성의 차이는 Table 3과 같다. 본 연구에서 자살 1회 시도자의 의식상태는 명료가 51명(40.8%)으로 가장 많았고, 다음은 기면 46명(36.8%), 혼미와 반혼수 26명(20.8%), 혼수 2명(1.6%)이었다. 자살 재시도자의 의식상태는 명료가 33명(47.1%)으로 가장 많았고, 다음은 기면 24명(34.3%), 혼미 및 반혼수 11명(15.7%), 혼수 2명(2.9%) 순으로 나타나 두 군간 통계적으로 유의한 차이가 없었다(χ^2^ = 1.46, *p* = .693). 본 연구에서 활력징후 중 수축기 혈압(t = 0.90, *p* = .513), 확장기 혈압(t = 0.50, *p* = .380), 심박동 수(t = −0.72, *p* = .164), 호흡수(t = −2.68, *p* = .094), 체온(t = −0.37, *p* = .391)과 산소포화도(t = 1.23, *p* = .246)는 두 군간 통계적으로 유의한 차이가 없었다. 본 연구에서 자살 1회 시도자의 KTAS 평균 등급은 2.36 ± 0.96이며 자살 재시도자는 2.34 ± 0.97으로 두 군간에 통계적으로 유의한 차이가 없었다(t = 0.13, *p* = .994) (Table 3).

### 4. 자살 1회 시도자와 자살 재시도자의 치료 관련 특성의 차이

자살 1회 시도자와 자살 재시도자의 치료 관련 특성의 차이는 Table 4와 같다. 본 연구에서 급성약물중독 치료 방법 중 기도삽관(χ^2^ = 1.59, *p* = .207), 활성탄(χ^2^ = 2.01, *p* = .156), 위세척(χ^2^ = 1.01, *p* = .316). 해독제(χ^2^ = 1.20, *p* = .273)는 두 군간 통계적으로 유의한 차이가 없었다. 본 연구에서 치료결과는 자의 퇴원, 병실 입원, 귀가, 중환자실 입원, 전원으로 구분하였으며, 두 군간 통계적으로 유의한 차이가 없었다(χ^2^ = 2.99, *p* = .559).

본 연구에서 혈액검사 중 자살 1회 시도자의 ANC는 6.12 ± 3.97×10^3^/μL이며, 자살 재시도자는 5.02 ± 3.18×10^3^/μL로(t = 1.97, *p* = .047), 자살 1회 시도자의 CRP는 13.08 ± 44.88 mg/L이고, 자살 재시도자는 4.53 ± 13.95 mg/L로(t = 1.55, *p* = .006), 자살 1회 시도자의 ALT는 25.61 ± 19.84 U/L, 자살 재시도자는 34.99 ± 65.25 U/L로 (t = −1.48, *p* = .009), 자살 1회 시도자의 CK는 256.42 ± 483.44 U/L, 자살 재시도자는 168.64 ± 241.32 U/L로 (t = 1.68, *p* = . 020), 자살 1회 시도자의 CK-MB는 4.86 ± 12.67 ng/mL이며, 자살 재시도자는 2.29 ± 4.34 ng/mL로 (t = 2.06, *p* = .011). BUN (t = 2.63, *p* = .005)은 두 군간에 통계적으로 유의한 차이가 있었다.

자살 1회 시도자의 WBC (t = 1.99, *p* = .188), 자살 1회 시도자의 RBC (t = 0.24, *p* = .077), ALP (t = 0.84, *p* = .460), AST (t = -0.47, *p* = .219), GGT (t = 0.47, *p* = .582), NT-proBNP (t= 0.68, *p* = .343). Creatinine의 평균은 0.77 ± 0.27이고, 자살 1회 시도자는 0.80 ± 0.28 mg/dL이고, 자살 재시도자는 0.70 ± 0.23 mg/dL로 두 군간 통계적으로 유의한 차이가 없었다(t = 2.59, *p* = .166).

본 연구에서 Chest X-ray 촬영(χ^2^ = 0.58, *p* = .445)과 심전도 결과(χ^2^ = 0.68, *p* = .409)를 정상, 비정상으로 구분한 결과는 두 군간에 통계적으로 유의한 차이가 없었다. 독성물질검사에서 검출된 독성물질은 자살 1회 시도자는 수면제가 84명(67.2%)으로 가장 많았고, 다음은 항우울제 41명(32.8%) 순이었다. 자살 재시도자는 수면제 45명(64.3%), 항우울제 24명(34.3%), 진통제 1명(1.4%)으로 두 군간 통계적으로 유의한 차이가 없었다(χ^2^ = 0.68, *p* = .409) (Table 4).

### 5. 자살 재시도에 영향을 미치는 요인

자살 재시도에 영향을 미치는 요인은 Table 5와 같다. 급성약물중독 자살 재시도에 영향을 미치는 요인을 규명하기 위해 자살 1회 시도자와 자살 재시도 간에 유의한 차이가 있는 것으로 나타난 정신건강의학과 질환, 중독 계절, 연령을 변수로 투입하여 로지스틱 회귀분석을 실시하였다. 분석 결과 정신건강의학과 질환을 진단받은 대상자가 진단받지 않은 대상자보다 자살 재시도가 1.99배 증가하는 것으로 나타났으며(odds ratio [OR]:1.99, 95% confidence interval [CI] = 1.04~3.80), 중독 계절은 봄보다 가을에 자살 재시도가 3.35배 증가하는 것으로 나타났으며(OR:3.35, 95% CI = 1.28~8.75), 연령은 18세 이하를 기준으로 65세 이상이 자살 재시도가 12.96배 많은 것으로 나타났다(OR:12.96, 95% CI = 2.02~83.17). 회귀모형은 유의한 것으로 나타났고(χ^2^ = 30.01, *p* < .001), 모형의 설명력을 나타내는 Cox와 Snell의 결정계수(R2)는 12.0%로, Nagelkerke의 결정계수(R2)도 17.0%의 설명력을 나타내었다. 이들 3개 요인이 급성약물중독 자살 재시도를 예측하는 정확성은 67.2%이었다.

## 논의

본 연구는 응급의료센터에 급성약물중독으로 내원한 자살시도자의 자살 재시도에 영향을 미치는 요인을 규명하기 위해 시도하였다. 응급의료센터에 급성약물중독으로 내원한 자살시도자의 특성을 파악하기 위해 응급의료센터에 급성약물중독으로 내원한 자살 1회 시도자와 자살 재시도자 간의 일반적 특성, 급성약물중독 관련 특성, 중증도 관련 특성, 치료 관련 특성, 자살 시도 관련 특성을 확인하고 자살 재시도에 영향을 미치는 요인이 정신질환 유무, 계절, 연령임을 확인하였다.

본 연구에서 응급의료센터에 내원한 35,424명 중 급성약물중독으로 내원한 대상자는 416명(1.2%)이었다. 이는 중앙응급의료센터[7]가 전국 응급실을 대상으로 급성약물중독으로 내원한 환자의 비율을 2.4%∼3.0%로 보고한 결과보다 낮았다. 본 연구는 특례시에 소재하는 대학병원의 응급의료센터를 내원한 환자를 대상으로 하였기 때문에 전국 단위로 조사한 중앙응급의료센터[7]에서 보고한 결과보다 낮은 것으로 생각한다. 본 연구에서 급성약물중독으로 내원한 416명 중 의도적으로 약물을 복용한 195명 중에서 자살을 재시도한 대상자는 70명으로 자살 재시도율은 35.9%이었다. 이는 Kim [6]이 자살시도로 응급실을 방문하고 자의 퇴원한 환자의 자살 재시도율을 56.8%로 보고한 결과보다 낮았다. 이러한 차이는 본 연구는 급성약물중독으로 응급실을 내원한 환자를 대상으로 하였으나 Kim [6]은 급성약물중독으로 내원한 환자 중 자의 퇴원한 환자를 대상으로 하였기 때문으로 생각한다. 이는 자의 퇴원한 환자의 자살 재시도율이 더 높은 것을 의미한다. 또한 Lee [22]의 연구에 의하면 자살시도 후 정신건강의학과 추적 진료를 받은 대상자의 자살시도 재발률이 낮았다. 이와 같은 본 연구와 선행연구[6,22]의 결과를 근거로 급성약물중독으로 자살을 시도하여 응급의료센터를 내원한 대상자의 자살 재시도를 예방하기 위해 대상자가 응급처치를 받은 후 자의 퇴원으로 귀가하지 않고 정신건강의학과 추적 진료를 받을 수 있도록 설명하거나 상담하는 체계를 구축하는 것이 필요하다. 현재 국가에서는 자살시도자의 사후관리를 위해 응급의료센터 기반 자살시도자 사후관리 사업을 시범 운영하고 있다. 그러나 이 시범 사업은 응급의료센터 내에 상주하는 정신건강 전문요원이 환자에게 상담받을 것을 권유하고 이를 수락한 환자에게만 사후관리를 실시하고 있어 참여하는 환자가 제한적이다[14]. 그러므로 응급의료센터에서 1차적으로 급성약물중독과 관련된 신체적인 건강문제를 해결하는데 적극 참여한 간호사의 자살에 대한 이해도를 높인다면, 자살시도자가 자살을 재시도하지 않도록 관리하는데 더 효율적일 것으로 생각된다.

본 연구에서 급성약물중독으로 응급의료센터를 내원한 자살시도의 자살 재시도에 영향을 미치는 요인은 정신건강의학과 질환, 중독 계절, 연령이었다. 응급의료센터에 내원한 급성약물중독 자살시도자를 대상으로 자살 재시도에 영향을 미치는 요인을 규명한 연구가 없어 자살 재시도자에 대한 선행연구 결과와 비교하고자 한다. 본 연구에서 정신건강의학과 질환 유무가 자살 재시도에 영향을 미치는 요인으로 규명되었는데 이는 Yun과 Jeon [14]은 정신건강의학과적 질환 유무를 자살 재시도에 영향을 미치는 요인으로 보고한 결과와 일치한다. 또한 Kim [6]과 Lee [22]의 연구에서 정신건강의학과 질환을 자살시도에 영향을 미치는 요인으로 보고한 결과와 유사하다. 이는 정신건강의학과 질환을 가지고 있으면서 자살을 시도한 과거력이 있는 환자는 자살을 재시도할 위험이 높음을 의미한다. 그러므로 정신건강의학과 질환을 가지고 있으면서 자살을 시도하여 응급실을 내원한 환자를 위한 자살 예방 대책을 마련하는 것이 필요하다. 본 연구에서 연령이 자살 재시도의 영향요인으로 규명되었으며 기준인 18세 이하보다 65세 이상에서 자살 재시도가 12.96배 높은 것으로 나타났다. 이는 Parra-Uribe 등[2]이 5년간 자살시도로 대학병원 응급의료센터를 내원한 환자의 자살 재시도를 조사한 연구에서 연령을 자살 재시도에 영향을 미치는 요인으로 보고한 연구와 유사하였다. 본 연구와 선행연구[2] 결과는 65세 이상 노인은 자살 재시도 위험이 높다는 것을 의미함으로 노인 자살과 자살 재시도를 예방하기 위한 사회적 차원의 관심과 지지가 필요하다.

본 연구에서 급성약물중독 자살시도자로 응급의료센터를 내원한 대상자의 성별은 여성이 남성보다 2배 높았다. 이는 보건복지부의 자살예방백서[1]에 응급실에 내원한 자살 시도자는 여성이 남성보다 1.54배 많은 것으로 보고한 결과와 유사하다. 국내뿐 아니라 국외 연구에서도 자살은 남성보다 여성에서 더 많이 시도하는 것으로 보고되어[22,23] 본 연구 결과와 일치한다. 이와 같은 연구결과는 Kim 등[24]이 남성에 비해 여성이 우울감의 평균이 높고, 다수의 여성이 겪는 임신, 출산, 폐경과 사회적인 요인으로 육아, 가사 노동의 병행 등으로 정신 건강 문제와 가족 문제가 있다고 보고한 결과로 설명할 수 있다.

본 연구 대상자의 일반적 특성 중 자살 1회 시도자와 자살 재시도자간에 차이가 있는 변수를 중심으로 살펴보면 기저질환 중 정신건강의학과 질환 유병률은 1회 시도자보다 자살 재시도자에서 유의하게 많았다. 이는 Kim 등[24]의 연구에서 자살시도자는 정신질환의 과거력이 있으며, 중독된 약물의 대다수가 정신건강의학과적인 약물로 보고한 결과와 일치하였다. 그러므로 정신건강의학과 질환을 가진 대상자들의 급성약물중독 재시도를 예방하기 위해 자살시도 과거력이 있거나, 급성약물중독 과거력이 있는 환자의 가족들은 환자의 정신건강의학과 치료 약물 복용 및 처방 등에 관심을 가지고 관리할 수 있도록 교육하는 것이 필요하다.

본 연구에서 급성약물중독물질은 정신건강의학과에서 처방받는 항우울제, 수면제 등 의약품이 가장 많은 비율을 차지하는 것으로 나타났고, 다음으로는 제초제, 살충제 등의 농약으로 나타나 선행연구[24]의 결과와 유사하였다. Lee 등 [25]이 의도적 중독으로 응급실을 방문한 환자의 특성을 분석한 연구에서 남성의 중독물질은 농약이 35.4%로 가장 많았으며, 여성은 수면제가 44.0%로 가장 많았고 그 다음이 농약이 14.2%순으로 보고한 결과와 유사하다. 본 연구에서 의약품 다음으로 급성약물중독 물질의 20%가 농약이었다. 이는 국가에서 농약의 유해성으로부터 인간과 생활환경을 보전하기 위해 농약관리법에 따라 고독성 농약의 판매를 규제하고, 농약을 안전하게 보관할 수 있도록 보관함을 보급하고 있으며, 구성성분의 변화 등 종합적인 관리체계를 갖추고 있음에도 불구하고 농약에 의한 급성약물중독 사고가 높은 비율로 발생하고 있으므로, 고독성 농약에 대한 관리 규제를 강화할 뿐 아니라 농약을 구매하는 자에 대한 교육 및 관리가 필요하다.

본 연구에서 급성약물중독 자살시도의 원인은 자살 1회 시도자 및 자살 재시도자 모두 우울, 불안과 같은 심리적 문제가 가장 많았으며, 다음으로는 가족 문제, 경제적 문제 등이 있었다. 자살 1회 시도자와 재시도자의 자살시도 원인을 비교한 연구가 없어 직접 비교할 수는 없어 자살시도자의 자살 동기와 비교하고자 한다. 보건복지부의 자살예방백서[1]에 따르면 자살시도자의 자살 동기는 남자의 경우 10대와 20대는 정신적 어려움, 30대∼50대는 경제적 어려움, 60대 이상은 육체적 어려움이 높았으며, 여자는 모든 연령대에서 정신적 어려움이 가장 높았다고 보고하여 본 연구 결과와 유사하였다. 이는 자살시도자의 자살시도 동기는 심리적 문제가 가장 큰 원인임을 의미한다. 응급의료센터에 급성약물중독으로 내원한 자살시도자를 최초로 간호하는 응급실 간호사는 자살시도자를 간호할 때, 급성약물중독과 관련된 신체적인 문제뿐 아니라 자살의 원인이 되는 문제를 해결할 수 있는 상담과 같은 심리 중재를 지속적으로 받을 수 있도록 정신건강의학과와 연계하는 것이 필요하다.

본 연구의 급성약물중독 독성물질은 수면제가 가장 많았으며, 다음으로 진통제, 항우울제 순이었으며, 정신건강의학과 질환자들이 자살을 시도할 때 주로 사용하는 중독물질은 치료 약물로 밝혀졌다. 이를 근거로 정신건강의학과 질환을 가진 환자가 치료약물을 복용하고 자살을 시도하여 응급의료센터를 내원한 후 자의 퇴원하는 경우에 정신건강의학과 치료의 필요성을 설명하고 지속적으로 정신건강의학과 치료를 받도록 권유함과 동시에 병원 내원이 필요한 위험증상을 교육하는 것이 필요하다. 또한 급성약물중독으로 내원한 대상자 뿐만 아니라 동거가족에게도 급성약물중독의 위험성 및 추후 관리에 대한 교육을 실시한다면 자살 재시도를 방지할 수 있을 것으로 판단된다.

본 연구에서 자살 1회 시도자와 자살 재시도자의 중증도는 2.34∼2.36 등급이었다. 이는 Ji 등[11]의 연구에서 응급실에 내원한 자살시도자의 KTAS의 평균이 2.55등급과 유사하였다. KTAS 2등급은 생명의 잠재적인 위협이 있으며 15분 이내 의사 진료 또는 간호사 재평가가 실시되어야 하는 긴급단계이다. 이는 본 연구와 선행연구[11]에서 자살을 시도하고 응급의료센터를 내원한 대상자의 중증도가 생명을 위협하는 단계와 응급 중재술이 필요한 정도의 중등도를 가지고 있음을 의미한다. 그러나 현재 국가에서 시행하고 있는 응급실 기반 자살시도자 사후관리사업은 자살시도자 중에서 사업에 참여하기를 희망하는 대상자에게 진료비를 지원하고 퇴원 후 전화 상담을 실시하는 사업이므로 응급실에 자살 시도 환자가 입원하였을 때 이를 관리할 수 있는 지침은 없다. 그러므로 응급의료센터를 내원한 대상자에게 신속하고, 적절한 응급처치를 하기 위해서는 급성약물중독으로 내원한 응급환자 관리에 대한 지침이 필요하다고 생각한다.

본 연구에서 자살 재시도자의 CK는 정상범위보다 높았다. 이는 Kim, Ku와 Hong [26]의 연구에서 자살시도자의 CK 수치가 정상범위보다 높았다고 보고한 결과와 유사하다. CK의 수치는 근육 또는 심장 세포가 손상된 경우 상승하기 때문에 급성약물중독이 근육이나 심장세포를 손상시켰음을 의미하므로[26] 급성약물중독 자살 재시도자를 위한 추후 관리에서 관리가 필요함을 의미하다.

본 연구는 급성약물중독으로 자살을 시도하고 응급의료센터를 내원한 환자의 의무기록을 중심으로 후향적으로 조사하였으며, 일개 대학병원의 응급의료센터를 대상으로 한 점, 코로나19가 유행한 2020∼2021년에 조사되었다는 점에서 연구결과를 일반화하는데 제한점이 있다. 그러나 본 연구는 급성약물중독으로 응급의료센터를 내원한 자살시도자를 1회 시도자와 재시도자로 구분하여 그 특성을 비교함으로써 급성약물중독으로 자살을 시도한 대상자들의 자살 재시도에 영향을 미치는 요인을 규명하였다는데 의의가 있다. 또한 본 연구 결과를 근거로 급성약물중독으로 자살을 시도하고 응급의료센터를 내원한 환자의 자살 재시도 위험도를 평가할 수 있는 체크리스트를 개발하는데 기초자료가 될 수 있는 자살 재시도 위험요인을 규명한 본 연구는 자살 시도자의 위험도를 평가하고, 위험도에 따라 자살 재시도 예방 간호 중재를 개발하는데 기초 자료를 마련하였다는 것에 간호학적 의의가 있다고 판단된다.

## 결론 및 제언

본 연구는 급성약물중독으로 응급의료센터에 내원한 자살시도자의 일반적 특성, 급성약물중독 관련 특성, 중증도 관련 특성, 치료 관련 특성을 파악하고, 자살 1회 시도자와 자살 재시도자의 특성을 비교하고, 자살 재시도에 영향을 미치는 요인을 규명하고자 시도하였다.

본 연구에서 급성약물중독으로 응급의료센터를 내원한 대상자의 자살 재시도에 영향을 미치는 요인은 정신건강의학과 질환 유무, 계절, 연령이었다. 본 연구 결과를 근거로 응급의료센터에 내원한 급성약물중독 자살시도자의 자살 재시도 위험을 파악할 수 있는 체크리스트를 개발하여 자살 시도자의 자살 재시도 위험도에 따른 예방 간호를 제공하는 것이 필요하다. 추후 자살 재시도를 예방할 수 있는 중재전략을 개발하기 위한 전향적 연구를 통해 자살 재시도에 영향을 미칠 수 있는 요인을 규명하고, 자살 재시도자의 경험을 심도 있게 이해하기 위해 질적 연구를 시도할 것을 제언한다.

## Figures and Tables

**Table 1.** Differences in the General Characteristics of Single Suicide Attempters and Suicide Reattempters (N = 195)

Variables	Characteristics		Single attempters (n = 125)	Suicide reattempters (n = 70)	t/χ^2^	*p*
			n (%)	n (%)		
Sex	Male		53(42.4)	22 (31.4)	2.28	.131
	Female		72(57.6)	48 (68.6)		
Age (yr)			42.15 ± 19.44	49.46 ± 19.13	-2.83	.625
Living situation	Alone		29 (23.2)	12 (17.1)	0.99	.319
	Living with family		96 (76.8)	58 (82.9)		
Occupation	Employed		23 (18.4)	15 (21.4)	0.26	.609
	Unemployed		102 (81.6)	55 (78.6)		
Underlying	HTN	Yes	17 (13.6)	9 (12.9)	0.02	.884
disease		None	108 (86.4)	61 (87.1)		
	DM	Yes	15 (12.0)	5 (7.1)	1.15	.284
		None	110 (88.0)	65 (92.9)		
	TB^†^	Yes	1 (0.8)	0 (0.0)	-	.641
		None	124 (99.2)	70 (100.0)		
	Liver disease^†^	Yes	0 (0.0)	1 (1.4)	-	.359
		None	125 (100.0)	69 (98.6)		
	Heart disease	Yes	4 (3.2)	0 (0.0)	2.28	.130
		None	121 (96.8)	70 (100.0)		
	Psychiatric diseases	Yes	53 (42.4)	43 (61.4)	6.50	.011
		None	72 (57.6)	27 (38.6)		

HTN = Hypertension; DM = Diabetes mellitus; TB = Tuberculosis.

† Fisher’s exact test.

**Table 2.** Differences in Characteristics related to Acute Drug Intoxication between Single Suicide Attempters and Suicide Reattempters (N = 195)

Variables	Characteristics	Single attempters (n = 125)	Suicide reattempters (n = 70)	χ^2^	*p*
		n (%)	n (%)		
Cause	Pessimism about disease	9 (7.2)	6 (8.6)	6.17	.290
	Family problems	40 (32.0)	16 (22.9)		
	Economic problems	23 (18.4)	10 (14.3)		
	Psychological problems	48 (38.4)	34 (48.6)		
	Interpersonal problems	4 (3.2)	1 (1.4)		
	Others	1 (0.8)	3 (4.2)		
Substance	Sedative-hypnotics	35 (28.0)	27 (38.6)	9.29	.158
	Antidepressant	45 (36.0)	30 (42.9)		
	Analgesic	8 (6.4)	4 (5.7)		
	Herbicide	25 (20.0)	7 (10.0)		
	Insecticide	6 (4.8)	1 (1.4)		
	Others	5 (4.0)	0 (0.0)		
	Unknown	1 (0.8)	1 (1.4)		
Place of suicide attempt	Inside the home	113 (90.4)	61 (87.1)	0.50	.482
	Outside the home	12 (9.6)	9 (12.9)		
Season of suicide attempt	Spring (March to May)	27 (21.6)	18 (25.7)	9.28	.026
	Summer (June to August)	31 (24.8)	20 (28.6)		
	Fall (September to November)	42 (33.6)	10 (14.3)		
	Winter (December to February)	25 (20.0)	22 (31.4)		
Time of suicide attempt	00:00 to 05:59	24 (9.2)	8 (11.4)	1.98	.576
	06:00 to 11:59	26 (20.8)	16 (22.9)		
	12:00 to 17:59	32 (25.6)	20 (28.6)		
	18:00 to 23:59	43 (34.4)	26 (37.1)		
Time of visiting the hospital	00:00 to 05:59	29 (23.2)	14 (20.0)	1.60	.659
	06:00 to 11:59	20 (16.0)	16 (22.9)		
	12:00 to 17:59	37 (29.6)	21 (30.0)		
	18:00 to 23:59	39 (31.2)	19 (27.1)		
Type of hospital visited	119 Ambulance	98 (78.4)	52 (74.3)	1.31	.519
	Other ambulance	7 (5.6)	7 (10.0)		
	Private car	20 (16.0)	11 (15.7)		
Route of visiting the hospital	Outpatient department	2 (1.6)	1 (1.4)	0.82	.663
	Hospital transfer	8 (6.4)	7 (10.0)		
	Visit to emergency room	115 (92.0)	62 (88.6)		

**Table 3.** Differences in Characteristics related to Severity between Single Suicide Attempters and Suicide Reattempters (N = 195)

Variables	Characteristics	Single attempters (n = 125)	Suicide reattempters (n = 70)	t/χ^2^	*p*
		n (%) or M ± SD	n (%) or M ± SD		
Consciousness status	Alert	51 (40.8)	33 (47.1)	1.46	.693
	Stupor	46 (36.8)	24 (34.3)		
	Semicoma	26 (20.8)	11 (15.7)		
	Coma	2 (1.6)	2 (2.9)		
Vital signs	SBP (mmHg)	125.70 ± 24.06	123.60 ± 24.87	0.90	.513
	DBP (mmHg)	74.83 ± 13.69	73.84 ± 13.69	0.50	.380
	HR (per min)	85.10 ± 16.19	87.09 ± 19.44	-0.72	.164
	RR (per min)	16.34 ± 1.37	16.93 ± 1.53	-2.68	.094
	BT (℃)	36.65 ± 0.51	36.69 ± 0.62	-0.37	.391
	SpO_2_ (%)	98.05 ± 2.40	97.34 ± 4.43	1.23	.246
KTAS	1	18 (14.4)	10 (14.3)	0.88	.831
	2	50 (40.0)	27 (38.6)		
	3	38 (30.4)	25 (35.7)		
	4	19 (15.2)	8 (11.4)		
		2.36 ± 0.96	2.34 ± 0.97	0.13	.994

M = Mean; SD = Standard deviation; SBP = Systolic blood pressure; DBP = Diastolic blood pressure; HR = Heart rate; RR = Respiration rate; BT = Body temperature; SpO2 = Saturation of percutaneous oxygen; KTAS = Korean triage and acuity scale.

**Table 4.** Differences in Treatment-related Characteristics between Single Suicide Attempters and Suicide Reattempters (N = 195)

Variables	Characteristics		Single attempters (n = 125)	Suicide reattempters (n = 70)	t/χ^2^	*p*
			n (%) or M ± SD	n (%) or M ± SD		
Treatment	Intubation	Yes	9 (7.2)	2 (2.9)	1.59	.207
		No	116( 92.8)	68 (97.1)		
	Charcoal	Yes	65 (52.0)	29 (41.4)	2.01	.156
		No	60 (48.0)	41 (58.6)		
	Gastric lavage	Yes	33 (26.4)	14 (20.0)	1.01	.316
		No	92 (73.6)	56 (80.0)		
	Antidote	Yes	10 (8.0)	9 (12.9)	1.20	.273
		No	115 (92.0)	61 (87.1)		
Result of	Discharge		19 (15.2)	11 (15.7)	2.99	.559
treatment	Self-discharge		52 (41.6)	37 (52.9)		
	General ward		32 (25.6)	12 (17.1)		
	Intensive care unit		18 (14.4)	8 (11.4)		
	Transfer		4 (3.2)	2 (2.9)		
	Death		0 (0.0)	0 (0.0)		
Chest radiography		Normal	94 (75.2)	56 (80.0)	0.58	.445
		Abnormal	31 (24.8)	14 (20.0)		
Echocardiography		Normal	54 (43.2)	26 (37.1)	0.68	.409
		Abnormal	71 (56.8)	44 (62.9)		
Toxicity	Sedative-hypnotics	Positive	84 (67.2)	45 (64.3)	1.87	.392
materials test		Negative	41 (32.8)	25 (35.7)		
	Antidepressant	Positive	41 (32.8)	24 (34.3)	1.63	.201
		Negative	84 (67.2)	46 (65.7)		
	Analgesic	Positive	0 (0.0)	26 (37.4)	0.01	.928
		Negative	125 (100.0)	44 (62.6)		
Hematologic test	WBC (10³/μL)		8.98 ± 4.07	7.91 ± 3.30	1.99	.188
	RBC (10^6^/μL)		4.45 ± 0.69	4.43 ± 0.51	0.24	.077
	ANC (×10^3^/μL)		6.12 ± 3.97	5.02 ± 3.18	1.97	.047
	CRP (mg/L)		13.08 ± 44.88	4.53 ± 13.95	1.55	.006
	ALP (U/L)		75.57 ± 38.03	71.43 ± 28.89	0.84	.460
	AST (U/L)		36.21 ± 40.12	40.68 ± 71.66	-0.47	.219
	ALT (U/L)		25.61 ± 19.84	34.99 ± 65.25	-1.48	.009
	GGT (U/L)		51.06 ± 87.55	43.96 ± 106.45	0.47	.582
	CK (U/L)		256.42 ± 483.44	168.64 ± 241.32	1.68	.020
	CK-MB (ng/mL)		4.86 ± 12.67	2.29 ± 4.34	2.06	.011
	NT-proBNP (pg/mL)		95.34 ± 185.54	78.66 ± 150.08	0.68	.343
	BUN (mg/dL)		15.26 ± 8.32	12.41 ± 4.66	2.63	.005
	Creatinine (mg/dL)		0.80 ± 0.28	0.70 ± 0.23	2.59	.166

M = Mean; SD = Standard deviation; WBC = White blood count or cell; RBC = Red blood count or cell; ANC = Absolute neutrophil count; CRP = C-reactive protein; ALP = Alkaline phosphatase; AST = Aspartate trasaminase; ALT = Alanine aminotransferase; GGT = Gamma-glutamyl transferase; CK = Creatine kinase; CK-MB = Creatine kinase–muscle brain; NT-proBNP = N-terminal pro b-type natriuretic peptide; BUN = Blood urea nitrogen.

**Table 5.** Factors Affecting Repeated Suicide Attempts (N = 195)

Independent variables	B	SE	*p*	OR	95% CI
Constant	-1.93	0.91	.034		
Psychiatric diseases					
None	-	-	-	Ref	
Yes	0.69	0.33	.038	1.99	1.04~3.80
Season of suicide attempt					
Spring	-	-	-	Ref	
Summer	0.21	0.45	.640	1.23	0.51~3.00
Fall	1.21	0.49	.014	3.35	1.28~8.75
Winter	-0.15	0.44	.733	0.86	0.36~2.05
Age (yr)					
≤ 18	-	-	-	Ref	
19–44	1.81	0.88	.039	6.11	1.10~34.06
45–64	1.91	0.88	.030	6.72	1.20~37.65
≥ 65	2.56	0.95	.007	12.96	2.02~83.17
χ^2 ^= 30.01, *p * < .001, Cox & Snell’s R^2^ = .12, Nagelkerke’s R^2^ = .17					

SE = Standard error; OR = Odds ratio; CI = Confidence interval; Ref = Reference.

## Data Availability

Please contact the corresponding author for data availability.
